# Rapid and non-destructive classification of rice seeds with different flavors: an approach based on HPFasterNet

**DOI:** 10.3389/fpls.2024.1502631

**Published:** 2025-01-20

**Authors:** Helong Yu, Zhenyang Chen, Shaozhong Song, Chunyan Qi, Junling Liu, Chenglin Yang

**Affiliations:** ^1^ Smart Agriculture Research Institute, Jilin Agricultural University, Changchun, China; ^2^ College of Information Technology, Jilin Agricultural University, Changchun, China; ^3^ School of Data Science and Artificial Intelligence, Jilin Engineering Normal University, Changchun, China; ^4^ Rice Research Institute, Jilin Academy of Agricultural Sciences, Changchun, China

**Keywords:** rice seed classification, japonica rice, deep learning, different flavored rice, lightweight network

## Abstract

Rice is an important part of the food supply, its different varieties in terms of quality, flavor, nutritional value, and other aspects of the differences, directly affect the subsequent yield and economic benefits. However, traditional rice identification methods are time-consuming, inefficient, and prone to damage. For this reason, this study proposes a deep learning-based method to classify and identify rice with different flavors in a fast and non-destructive way. In this experiment, 19 categories of japonica rice seeds were selected, and a total of 36735 images were finally obtained. The lightweight network High Precision FasterNet (HPFasterNet) proposed in this study combines the Ghost bottleneck and FasterNet_T0 and introduces group convolution to compare the model performance. The results show that HPFasterNet has the highest classification accuracy of 92%, which is 5.22% better than the original model FasterNet_T0, and the number of parameters and computation is significantly reduced compared to the original model, which is more suitable for resource-limited environments. Comparison with three classical models and three lightweight models shows that HPFasterNet exhibits a more comprehensive and integrated performance. Meanwhile, in this study, HPFasterNet was used to test rice with different flavors, and the accuracy reached 98.98%. The experimental results show that the network model proposed in this study can be used to provide auxiliary experiments for rice breeding and can also be applied to consumer and food industries.

## 1 Introduction

As the staple food for nearly half of the world’s population, rice provides a rich source of nutrients (Verma and Srivastav, 2020), and its yield and quality are important for global food security, soil conservation, and genetic diversity (Verma et al., 2021). There are significant differences in the quality of different varieties of rice, and these differences are mainly affected by natural factors such as climate, soil, and water, as well as by human factors such as planting technology and variety selection (Lu et al., 2019). Generally speaking, rice with good quality is more likely to be favored by the market, thus bringing higher economic benefits (Creppy et al., 2024). In many countries, the production and sale of seeds have been commercialized, with huge economic profits lurking (Qaim, 2020). Poor-quality rice is often exploited by unscrupulous traders to counterfeit high-quality varieties on the market for higher profits. However, traditional methods of rice variety identification are often time-consuming, expensive, and usually only available for small batches (Koklu et al., 2021a). Computer vision and deep learning are popular for being non-contact, non-destructive, and inexpensive, which can automatically extract the features of rice images, quickly process a large amount of data, and realize accurate classification through algorithmic models, which significantly improves the classification efficiency and accuracy (Zareiforoush et al., 2015). Therefore, the appearance characteristics of rice seeds are crucial to the accuracy of computer vision recognition (Patrício and Rieder, 2018).

In the past, many scholars have conducted related research. Yufei Ge’s team proposed a real publicly available benchmark dataset for the classification of rice seed hyper-spectral imaging systems. Meanwhile, they proposed a difficulty-weighted k-nearest neighbor-based algorithm, IDKNN, for the hyperspectral classification of rice seeds and achieved very excellent results (Ge et al., 2024). Baichuan Jin’s team, on the other hand, utilized NIR hyperspectral imaging in combination with deep learning to successfully differentiate between different varieties of rice seeds, in particular, they employed NIR-HSI with LeNet, GoogLeNet and residual network (ResNet) models for recognition, among which, the ResNet model has the best classification effect, and the classification accuracy of the test set reaches 86.08% (Jin et al., 2022). Deepa Joshi’s team achieved the label-free and lossless classification of rice seeds by deep neural network and optical coherence tomography with a good classification effect (Joshi et al., 2021). Hengnian Qi’s team utilized near-infrared hyperspectral imaging for the detection of rice seed viability and combined it with a transfer learning method to achieve significant results. They used the CNN model of Yongyou 12 constructed with MixStyle migration knowledge to classify the vigor of Yongyou 1540, Su Xiang Japonica 100, and Long Japonica 1212, and the accuracy reached 90.00%, 80.33%, and 85.00%, respectively, which was an excellent performance (Qi et al., 2023). Jinfeng Zhao’s team utilized the rotationally aware deep learning model YOLO-rot to measure the size of rice seeds and achieved remarkable results (Zhao et al., 2023). Chunguang Bi and other scholars proposed a seed classification model based on the Swin Transformer, which utilizes the self-attention mechanism to effectively extract image information, focuses on feature attention and multi-scale feature fusion learning, and demonstrates accurate and efficient ability to classify seeds. The MFSwin Transformer model achieved a remarkable average accuracy, recall, and F1 score of 96.53%, 96.46%, and 96.47%, respectively, on the test set with a parameter count of 12.83 M (Bi et al., 2022). Murat Koklu’s team developed a non-destructive model to improve the classification success rate by utilizing images of rice varieties for classification. In this model, they extracted 106 morphological and color features from rice images as inputs to artificial neural networks and DNNs and successfully performed classification (Koklu et al., 2021b). Helong Yu (Yu et al., 2024) proposed an improved residual network method based on the characteristics of rice to effectively classify rice seeds of the same variety grown in different regions, and the accuracy of the proposed model reaches 95.13%, which is an improvement of 7.56% to the original model and achieves very good results. Hongwei Li (Li et al., 2024) proposed and disclosed a dataset of dragon fruit, which was captured under different conditions, including multiple angles, different lighting conditions, and different weather conditions. The proposed enhanced YOLOv5s model exhibits an impressive 97.80% average accuracy and achieves an impressive 139 frames per second (FPS) in a GPU running environment. Compared to current state-of-the-art models, the improved YOLOv5 performs well and demonstrates the preferred level of overall performance. Mingyou Chen (Chen et al., 2024) proposed a set of vision algorithms for motion destination estimation, real-time self-localization and dynamic harvesting. In addition, a solid coordination mechanism for continuous motion and harvesting behavior is established. Each method has unique advantages, such as improving accuracy, adapting to different conditions, improving harvesting efficiency, enabling autonomous continuous operation of the robot, and validating the rationality of the methods in comprehensive field trials. To sum up, research in hyperspectral and near-infrared spectroscopy is costly and inefficient, making it difficult to realize large-scale applications. In addition, the performance of related studies based on deep learning models may decline when confronted with more rice varieties, and numerous scholars may not be able to balance the accuracy of the model and the parameters of the model itself.

In response to the above problem, this study proposes a fast, lossless, and inexpensive lightweight network to categorize different varieties of rice seeds. In the area of deep learning, standard convolution (Conv) has strong expressive ability and accuracy, and its strong expressive ability and accuracy are its core advantages (Cong and Zhou, 2023). However, since each convolutional kernel needs to learn multiple weight values and there is no shared weight between different convolutional kernels, the number of parameters of Conv is usually large (Tulbure et al., 2022). In this study, FasterNet (Chen et al., 2023) is chosen as an improved model mainly because FasterNet significantly reduces redundant computations and memory accesses by introducing the new technique of partial convolution (PConv), which makes FasterNet run very fast on a wide range of devices while maintaining a high level of accuracy. The Ghost module in GhostNet (Han et al., 2020)generates a large number of ghost feature maps through cheap operations and then undergoes a small number of conventional convolutions, thus enlarging the width of the network and improving the feature representation capability without increasing the computational effort.

1. The research content and methods of this paper include:
  Nineteen varieties of flavored rice seeds were collected and their RGB images were acquired. The images of individual rice seeds were obtained by image segmentation.
2. Combining Ghost bottleneck (Han et al., 2020) with FasterNet (Chen et al., 2023) while introducing group convolution to get HPFasterNet. A learning rate dynamic adjustment strategy is introduced during model training. Compared with the original model, HPFasterNet is more accurate, more efficient, and less weighted.
3. Evaluate the classification performance of the model and compare it with ResNet50 (He et al., 2016; Xie et al., 2017), ConvNeXt_T (Liu et al., 2022), RepVggNet_A1 (Ding et al., 2021), GhostNet (Han et al., 2020), ShuffleNet (Zhang et al., 2018) and MobileNetV2 (Sandler et al., 2018) models, respectively.
4. The changes in the results before and after the improvement were analyzed and the test results of four different flavored rice were analyzed using the improved model.

## 2 Materials and methods

### 2.1 Sample collection and preprocessing

**Figure 1:**
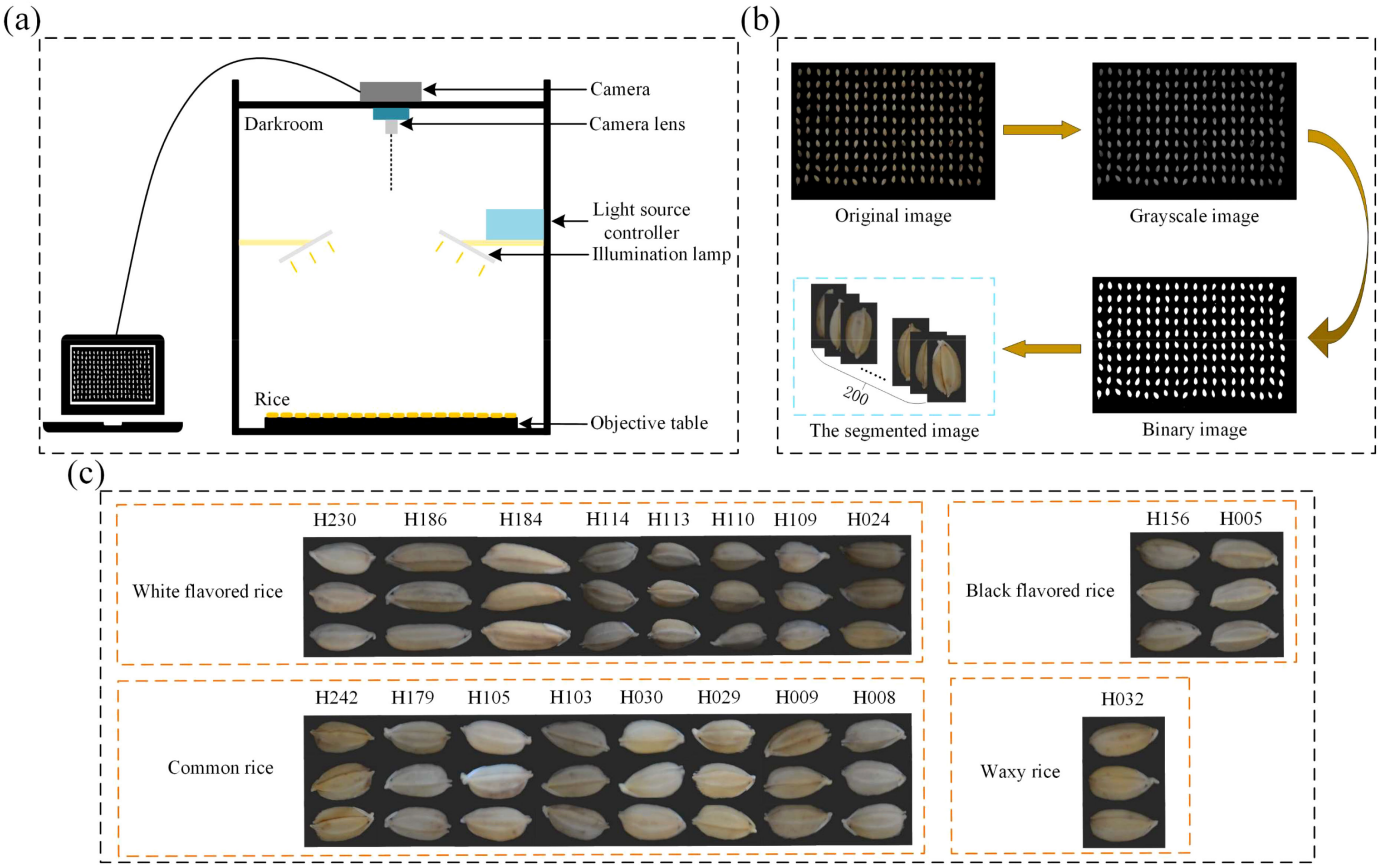
Schematic diagram of the data acquisition process, **(A)** is the schematic diagram of the imaging system, **(B)** is the schematic diagram of the process of threshold segmentation, and **(C)** is the demonstration of the effect of image segmentation.

All samples in this study were obtained from the Rice Research Institute of Jilin Academy of Agricultural Sciences, China. The sample for the study consisted of 19 varieties of japonica rice. The imaging system is shown in **Figure 1A** and consists of a NikonD7100 camera and a lens, a light control system controlling two lights, and at the bottom, a carrier for the seeds, which is wrapped in a black light-absorbing cloth and then shot vertically by the camera. During data collection, 200 rice seeds of the same variety were first randomly selected and arranged in a 10 × 20 grinding apparatus. Then, without seeds overlapping or sticking, they were inverted on a black cloth and their RGB images were acquired by the camera.

The process of data preprocessing is shown in **Figure 1B**. A threshold segmentation method (Al-Amri and Kalyankar, 2010), is used to select an appropriate threshold value to divide the pixels in the image into two categories (target and background), and in this study, the threshold value is set to 0.3, and all the values below 0.3 are set to 0 (black), and all the values above 0.3 are set to 1 (white), and the binary image is obtained based on the grayscale image. The binary image is then multiplied pixel-by-pixel with the original image so that only pixels in the mask image with a value of 1 (representing the target region) are preserved in the original image, while pixels with a value of 0 (representing the background region) are set to black. The edges of the rice seeds are then extracted based on the pixel distributions of the image with the background removed by utilizing the contour extraction algorithm, thus extracting the target region. As shown in **Figure 1C**, three target images are displayed for each variety, which is divided into four different flavors, and it can be seen that different varieties of rice seeds have very diverse morphologies. In this experiment, useless images need to be eliminated after photographing the seed grains, such as those that are blurred, damaged, or do not meet the experimental requirements at all, which may interfere with the training and evaluation process of the model and cause the model to learn wrong or irrelevant features.

As shown in **Table 1**, a total of 36735 images are segmented, and then the training set, validation set, and test set are randomly divided according to the ratio of 6:2:2. That is, 22035 images are used for training, 7348 images are used for validation, 7352 images are used for testing, and labeled accordingly.

**Table 1:** Profile of samples.

Label	Variety	Type of Taste	Training Set	Validation Set	Test Set
1	H005	Black flavored rice	1167	389	389
2	H008	Common rice	1171	391	391
3	H009	Common rice	1167	389	390
4	H024	White flavored rice	1167	389	389
5	H029	Common rice	1191	397	397
6	H030	Common rice	1192	398	398
7	H032	Waxy rice	1169	390	389
8	H103	Common rice	1176	392	393
9	H105	Common rice	1191	397	397
10	H109	White flavored rice	1059	353	353
11	H110	White flavored rice	1106	369	368
12	H113	White flavored rice	1045	348	349
13	H114	White flavored rice	1185	395	396
14	H156	Black flavored rice	1165	389	389
15	H179	Common rice	1143	381	381
16	H184	White flavored rice	1192	398	398
17	H186	White flavored rice	1181	394	394
18	H230	White flavored rice	1170	390	391
19	H242	Common rice	1198	399	400
/	Total	/	22035	7348	7352

### 2.2 Model building

#### 2.2.1 The three convolutions used for the experiment

**Figure 2:**
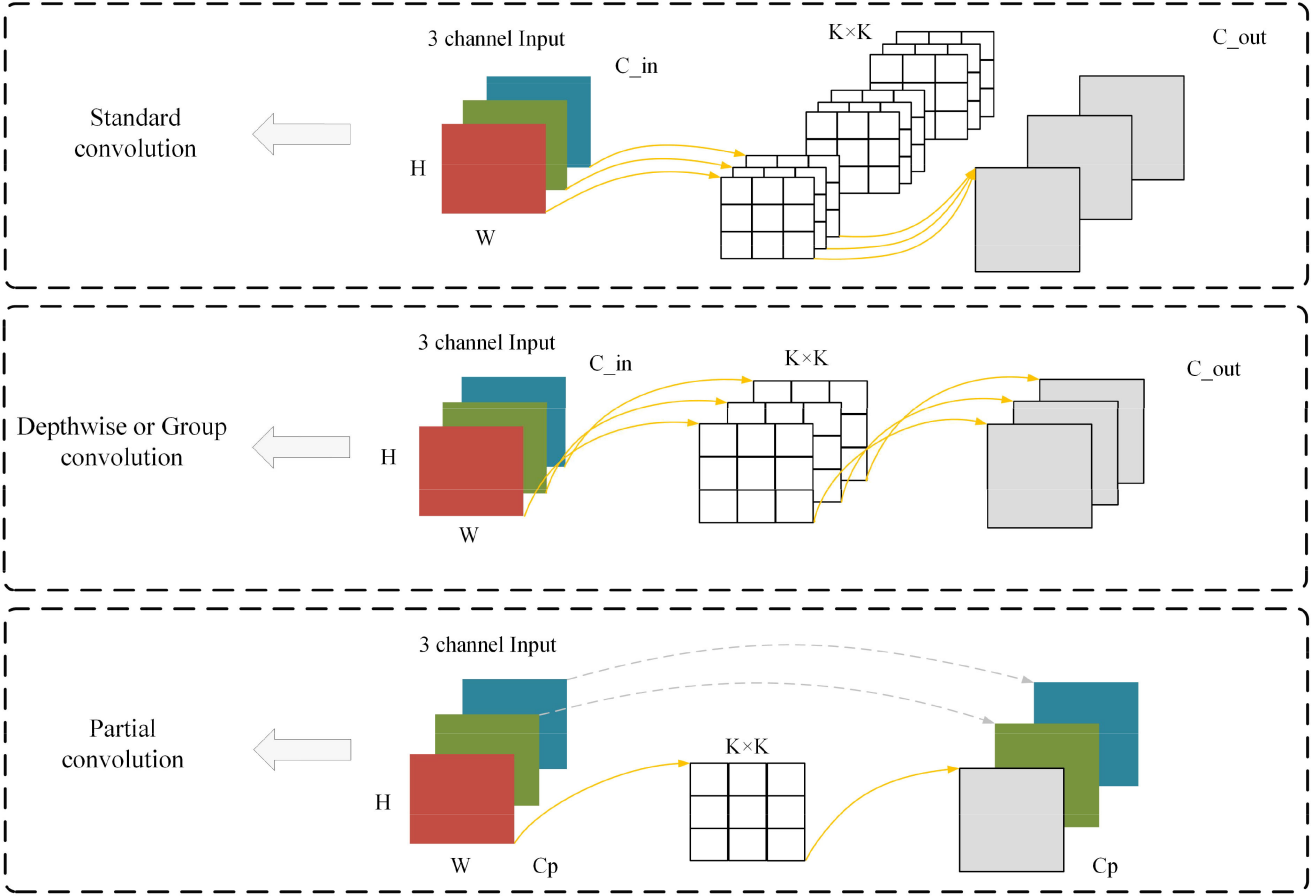
Schematic representation of the three convolutions in this study.

As shown in **Figure 2** is a schematic diagram of the three convolutions used in this study, namely Standard convolution(Conv), Depthwise Convolution(DWConv) or Group Convolution (GConv) (Cohen and Welling, 2016), and Partial Convolution(PConv) (Chen et al., 2023).

Depthwise Convolution/Group Convolution (DWConv/GConv) compensates for some of the drawbacks of Conv by drastically reducing the number of parameters. Since the convolution kernel of DWConv is only convolved for a single channel and does not share weights between different channels, the number of parameters is usually much smaller than that of Conv. This helps to reduce the risk of overfitting and makes the model easier to train. Due to the small number of parameters, DWConv is also faster to compute, so it can build lighter-weight neural network models and reduce the consumption of storage and computational resources, making it suitable for use in scenarios with limited computational resources, such as mobile devices or embedded systems. Meanwhile, since DWConv only performs spatial convolution independently for each channel, its representation ability may be weaker compared to Conv.

Partial Convolution (PConv) is from the model FasterNet, which argues that the main cause of high computation is frequent memory accesses. **Figure 2** expresses the design of PConv, which exploits redundancy in feature mapping and systematically applies the Conv on only a portion of the input channels, while the rest of the channels are left unchanged. In essence, PConv has lower FLOPs than Conv and higher FLOPs than DWConv. In other words, PConv makes better use of the computational power on the device, while PConv is also very efficient in extracting spatial features.

The parameters and FLOPs of these three convolutions are shown in **Table 2**, assuming that the number of input and output channels are $C_{\_in}$ and $C_{\_out}$ , respectively, $K^{2}$ is the kernel size of the convolution, *G* is the size of the group in DWConv/Gconv, and the size of the input data is *H×W*. $C_{p}$ is the number of channels used in PConv for spatial feature extraction, and the computation in PConv is only 1/16 of that of Conv in the case of a typical partial ratio of r= $C_{p}$ / $C_{\_in}$ =1/4.

**Table 2:** Comparison of parameters and FLOPs for Conv, DWConv/GConv, and PConv.

Name	Parameters	FLOPs
Conv	$K^{2}\times C_{\_in}\times C_{\_out}$	$C_{\_in}\times K^{2}\times H\times W\times C_{\_out}$
DWConv/GConv	$K^{2}\times \frac{C_{\_in}}{G}\times C_{\_out}$	$K^{2}\times \frac{C_{\_in}}{G}\times H\times W\times C_{\_out}$
PConv	$K^{2}\times C_{p}^{2}$	$K^{2}\times H\times W\times C_{p}^{2}$

#### 2.2.2 Ghost bottleneck

To improve the performance of the model, Ghost bottleneck was introduced in this experiment. The output feature maps of convolutional layers often contain much redundancy, and some of them could be similar to each other. The authors of GhostNet (Han et al., 2020) point out that it is unnecessary to generate these redundant feature maps one by one with a large number of FLOPs and parameters. Suppose that the output feature maps are “ghosts” of a handful of intrinsic feature maps with some cheap transformations. These intrinsic feature maps are often of smaller size and produced by ordinary convolution filters.

**Figure 3:**
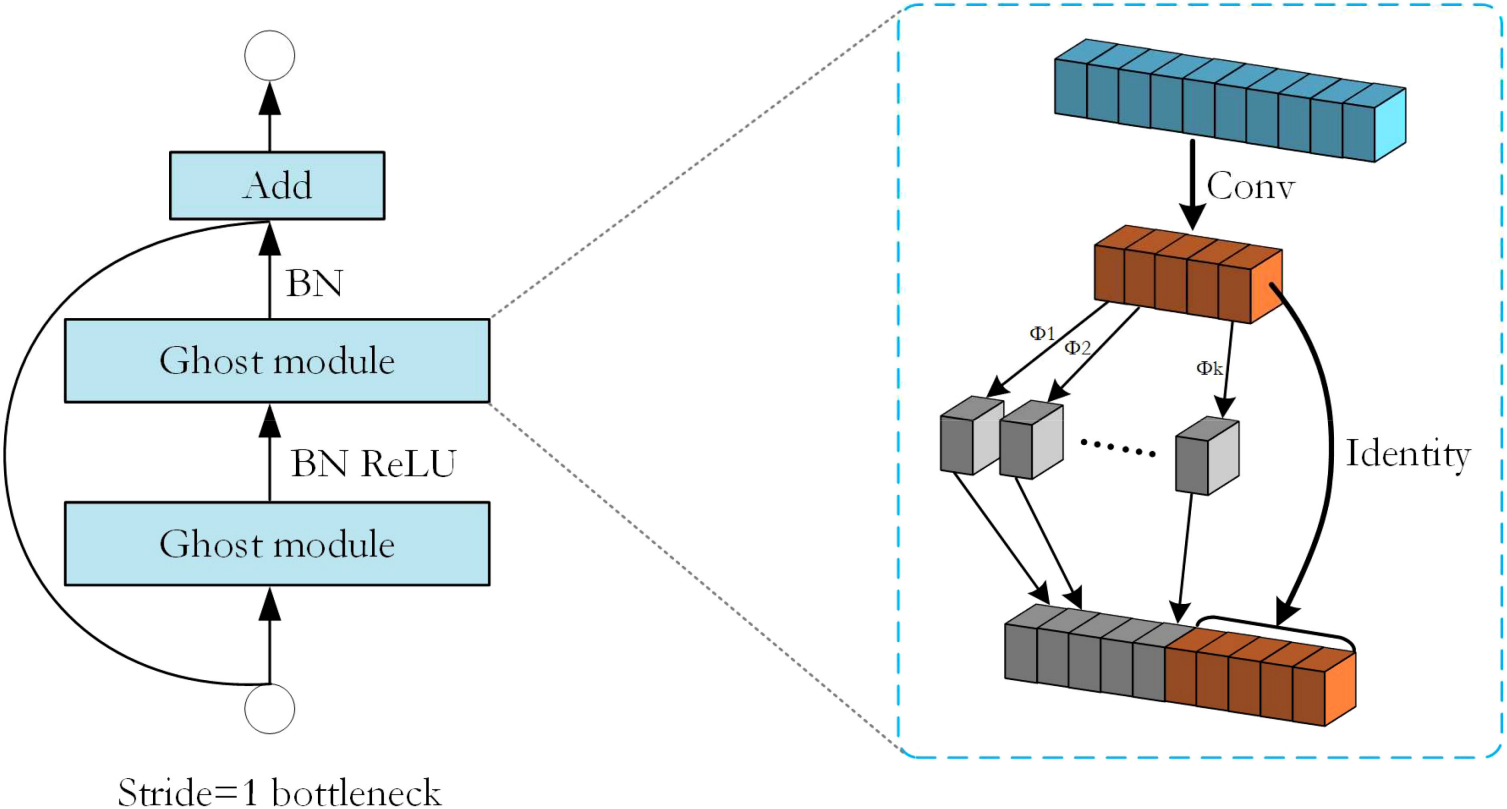
The structure of the Ghost bottleneck with Stride=1 and the structure of the Ghost module inside it.

Specifically, given the input data $X\in R^{\text{c}\times h\times \text{w}}$ , where c is the number of input channels and *h* and w are the height and width of the input data, *m* intrinsic feature maps $Y\in R^{{\text{h}}^{\prime }\times {\text{w}}^{\prime }\times \text{m}}$ are generated using a primary convolution:


(1) $$\begin{array}{c} Y=X*f \end{array}$$

where $f\in R^{\text{c}\times k\times k\times \text{m}}$ is the utilized filters, *m* is smaller than the output feature map with *n* channels, and ∗ is the convolution operation. In addition, ${\text{h}}^{\prime }$ and ${\text{w}}^{\prime }$ are the height and width of the output data, and k×k is the kernel size of convolution filters *f*. The hyper-parameters such as filter size, stride, and padding, are the same as those in the ordinary convolution to keep the spatial size ( ${\text{h}}^{\prime }$ and ${\text{w }}^{\prime }$ ) of the output feature maps consistently. To further obtain the desired *n* feature maps, GhostNet proposes to apply a series of cheap linear operations on each intrinsic feature in *Y* to generate *s* ghost features according to the following function:


(2) $$\begin{array}{c} y_{ij}=\Phi_{i,j}(y_{i}^{'}),\;\;\;\;\;\;\;\;\forall \;\;i=1,\ldots ,m,\;\;j=1,\ldots ,s \end{array}$$

where $y_{i}^{'}$ is the *i*-th intrinsic feature map in *Y*, $\Phi_{i,j}$ in the above function is the *j*-th (except the last one) linear operation for generating the *j*-th ghost feature map $y_{ij}$ , that is to say, $y_{i}^{'}$ can have one or more ghost feature maps. The last $\Phi_{i,s}$ is the identity mapping for preserving the intrinsic feature maps as shown in **Figure 3**. By utilizing Equation 2, we can obtain n = m · s feature maps *Y* = [ $y_{11}$ , $y_{12}$ , · · ·, $y_{ms}$ ] as the output data of a Ghost module as shown in **Figure 3**. Note that the linear operations Φ operate on each channel whose computational cost is much less than the ordinary convolution.

#### 2.2.3 GeLU activation function and ReLU activation function

The ReLU activation function is a simple and commonly used activation function that sets negative inputs to zero while positive inputs are held constant. Its formula is as follows:


(3) $$\begin{array}{c} f(x)=\{\begin{array}{c} x,x\geq 0\\ 0,x<0 \end{array} \end{array}$$

The ReLU activation function introduces a nonlinear property that allows the neural network to learn nonlinear relationships and thus better adapt to complex data patterns. The computation of the ReLU function is very simple, it only needs to compare the inputs and keep the positive values without complex mathematical operations, thus making it suitable for large-scale neural networks. Therefore, the computation is fast and suitable for large-scale neural networks. During training, the ReLU activation function can activate one part of the neurons and set the other part to zero, this sparsity helps to reduce the risk of overfitting and improve the generalization ability of the model.

The GeLU activation function is a smooth and approximate ReLU activation function that adds the properties of a Gaussian error function to ReLU. Its formula is as follows:


(4) $$\begin{array}{c} f(x)=0.5\times x\times (1+\tanh(\sqrt{\frac{2}{\pi }}\times (x+0.044715x^{3}))) \end{array}$$

The Tanh function is publicized as follows:


(5) $$\begin{array}{c} f(x)=\frac{e^{x}-e^{-x}}{e^{x}+e^{-x}} \end{array}$$

The GeLU activation function is a smooth curve with continuity and conductivity, which makes it easier to optimize the neural network during training. In most cases, the GeLU activation function is very close to the ReLU function, so it can retain most of the advantages of ReLU while having smoother properties. The GeLU activation function performs well with noisy data, and its properties based on the Gaussian error function can better deal with the uncertainty in the distribution of the data.

**Figure 4:**
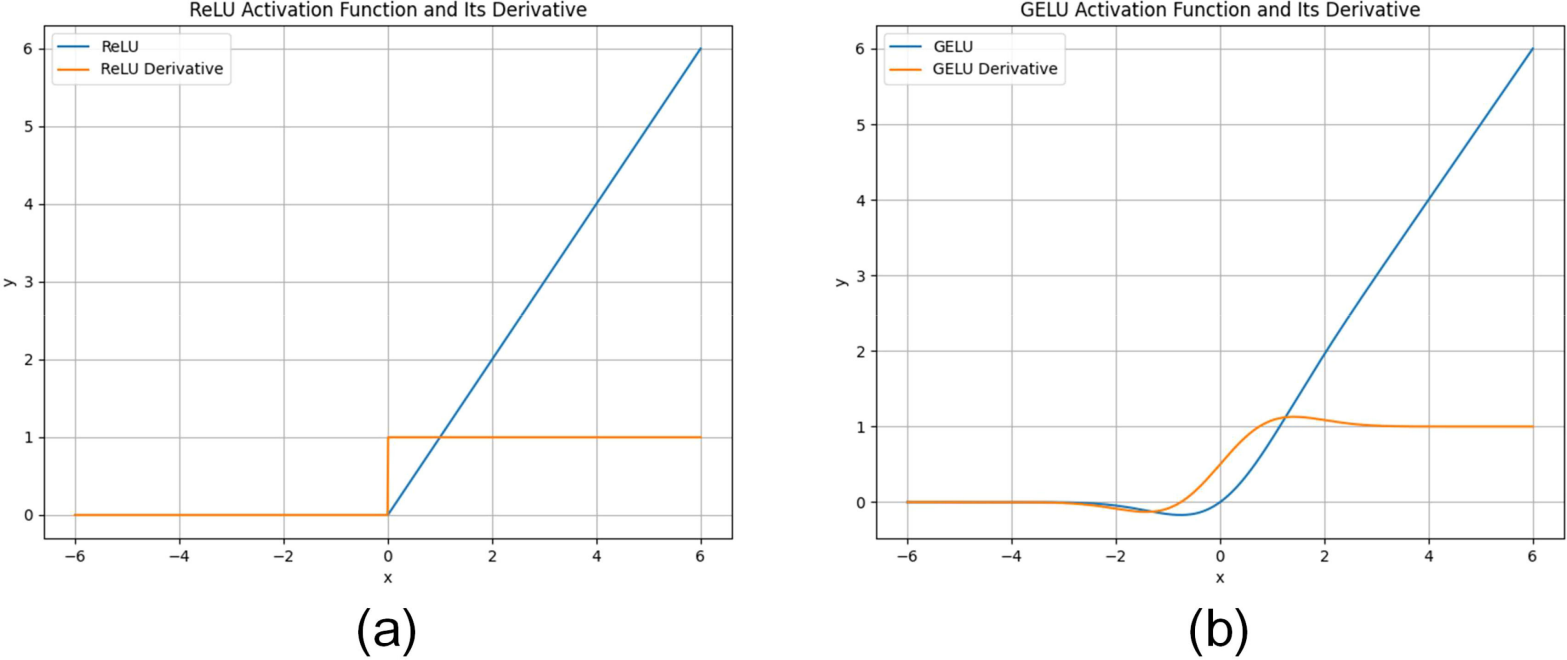
**(A)** shows the curves of the ReLU activation function and its derivative, and **(B)** shows the curves of the GeLU activation function and its derivative.

The ReLU activation function is simple and efficient for most deep learning tasks, while the GeLU activation function provides smoother properties while retaining the benefits of ReLU for scenarios that require better robustness. The curves of the ReLU activation function and the GeLU activation function, as well as a comparison of the curves of their derivatives, are shown in **Figure 4**.

#### 2.2.4 Cross Entropy Loss

The loss function used in this study is Cross Entropy Loss (Zadeh et al., 2020), Cross Entropy Loss is robust to the probability distribution predicted by the model. Even if the model has a small deviation in the predicted probability of some categories, it will not affect the overall loss too much. This makes the model more stable during training and less susceptible to noise or outliers. The binary classification Cross Entropy Loss is shown below:


(6) $$\begin{array}{c} L=\;-[y\log p+(1-y)\log(1-p)] \end{array}$$

Where y denotes the sample label and p denotes the probability that the corresponding sample label is predicted to be positive. In the multiclassification task, each sample may have more than one possible category, and the model output is the probability distribution of each sample belonging to each category, Cross Entropy Loss can measure the distance between the probability distribution of the model output and the true labels, to guide the model optimization. The multicategory Cross Entropy Loss formula is shown below:


(7) $$\begin{array}{c} L=\;-\sum_{c=1}^{M}y_{c}\log p_{c} \end{array}$$

where $p_{c}\;$ denotes the probability that the label is predicted to be c.

#### 2.2.5 FasterNet_T0 and HPFasterNet

**Figure 5:**
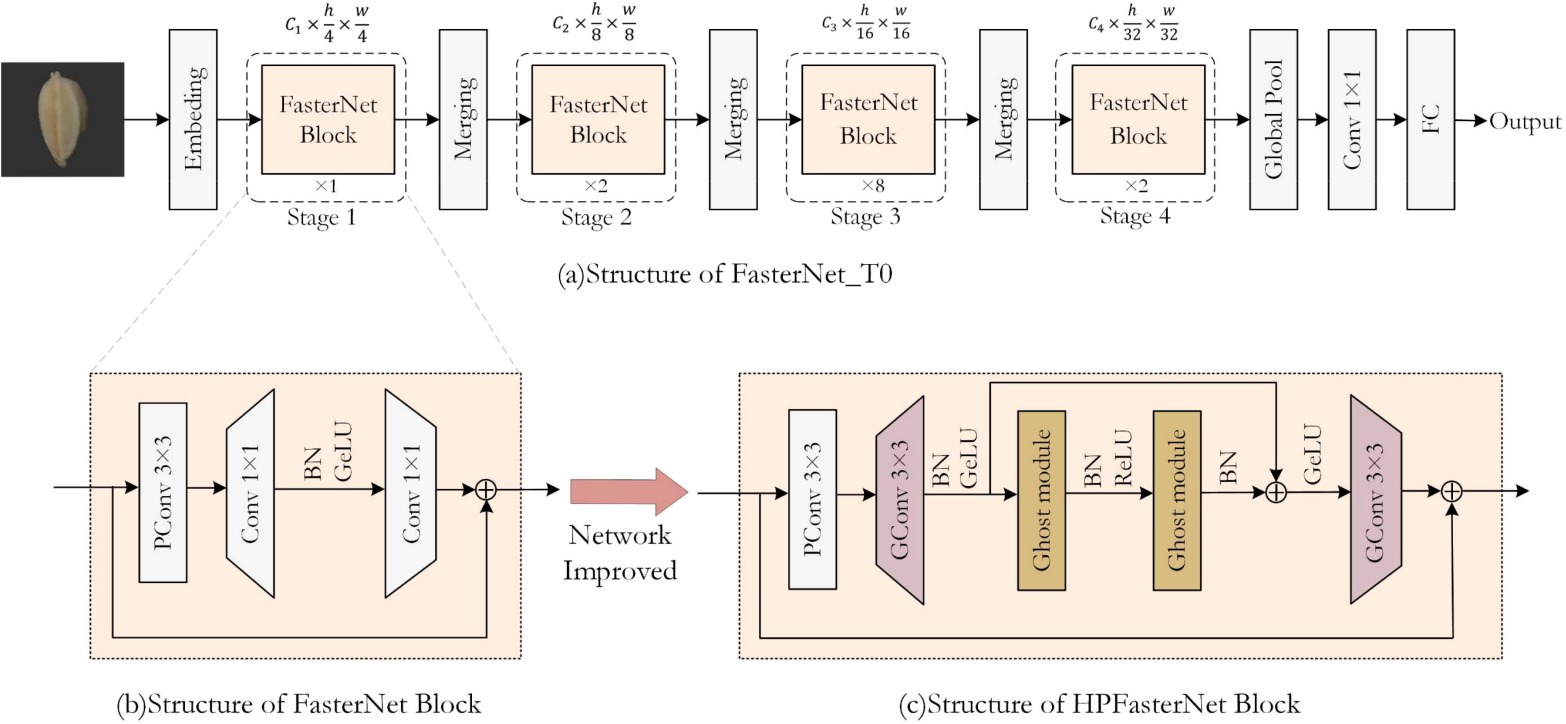
The overall architecture of FasterNet, the structure of FasterNet Block, and the schematic diagram of the modified HPFasterNet Block.

The improvement process of the model is shown in **Figure 5**. To improve the performance of the model and reduce the computation, the main purpose of this study is to replace the 1×1Conv used for upscaling and downscaling with 3×3GConv in the residual structure in FasterNet is to increase the nonlinearity while expanding the receptive field, reducing the number of parameters, and improving the computational efficiency. The 3×3GConv has a higher degree of nonlinearity compared to the 1×1Conv has a higher degree of nonlinearity, which helps the model to learn more complex features and patterns. The 3×3 convolutional kernel has a larger receptive field than the 1×1 convolutional kernel, which means it can capture a wider range of information. In deep neural networks, the size of the receptive field is critical for capturing spatial relationships in an image.

At the same time, this study synthesizes the advantages of FasterNet and GhostNet, and introduces the Ghost bottleneck into the residual structure, so that more and more complex features are extracted through the Ghost bottleneck for the channels that have been amplified. From the overall architecture of the model, it can be seen that the HPFasterNet block is structured as a double residual structure, and the residual block is still nested within the residual structure, and this structure makes the model much more accurate. Although this design leads to a slight increase in the computation and the number of parameters of the model to a certain extent, when introduced in combination with GConv, the number of parameters of the model will be reduced relative to the original model.

#### 2.2.6 Learning rate dynamic adjustment strategy in this study

If the learning rate is set too high or too low, it can have a significant impact on the learning process of the model (Croitoru et al., 2024). If the learning rate is set too high, the advantage is that the model may update the weights faster and thus explore the possible solution space faster. But then, the disadvantage is also obvious that the model may miss the optimal solution because the step size is too large, leading to oscillations or even divergence during the training process, making it difficult to converge to a stable solution. On the contrary, if the learning rate is set too low, although the model can converge more stably, the speed of convergence may be very slow, the advantage is that the model may adjust the weights more finely to get a more accurate solution, but the disadvantage is also obvious, the training process will become very time-consuming, and the model may be more likely to fall into the local optimal solution, and cannot find the global optimal solution. Therefore, it is very important to set the appropriate learning rate, so we choose the gradient decay strategy to dynamically adjust the learning rate during the training process. Its formula is as follows:


(8) $$\begin{array}{c} lr_{n}=initial_{lr}\;\times \;d\left[\frac{n-1}{p}\right] \end{array}$$

$lr_{n}$ is the learning rate of the *nth* round of model training, $initial_{lr}$ is the initial learning rate, *d* is the decay factor, d=0.85 in this study, *n* is the current round of model training, *p* stands for how many rounds decay once, p=4 in this study, $\left[\frac{n-1}{p}\right]$ represents the downward rounding of $\frac{n-1}{p}$ , i.e., to take the largest integer that is not larger than $\frac{n-1}{p}$ integer. In this study, it is called the “8-5 Gradient”.

### 2.3 Evaluation indexes of model

In the field of machine learning, confusion matrices are often used to compare the results of model classification in supervised learning. Take the binary classification problem as an example, define that the actual result is positive and the predicted result is positive, denoted as TP; if the actual result is negative, the predicted result is positive, denoted as FP; if the actual result is positive, the predicted result is negative, denoted as FN; if the actual result is negative, the predicted result is negative, denoted as TN.

Accuracy (Acc), Precision (P), Recall (R), and F1-score (F1) can be computed from the data in the confusion matrix and used as evaluation metrics for assessing the classification performance of the model.

Accuracy is the ratio of the number of positive and negative samples correctly predicted to the total number of samples, and its formula is:


(9) $$\begin{array}{c} Acc=\;\frac{TP+TN}{TP+FP+FN+TN} \end{array}$$

Precision is the ratio of the number of correctly predicted positive samples to the total number of samples predicted to be positive, and its formula is:


(10) $$\begin{array}{c} P=\;\frac{TP}{TP+FP} \end{array}$$

Recall is the ratio of the number of correctly identified positive samples to the total number of actual positive samples, and its formula is:


(11) $$\begin{array}{c} R=\;\frac{TP}{TP+FN} \end{array}$$

F1-score is the harmonic mean of precision and recall, and its formula is:


(12) $$\begin{array}{c} F1=\;\frac{2TP}{2TP+FP+FN} \end{array}$$

### 2.4 Training hyperparameter information and experimental environment configuration

This study provides information on the specific experimental parameters used in training the new network model proposed in this paper. In this study, the input size of the dataset is set to 224 × 224, the number of training rounds is 100, the base learning rate is set to 0.01, the Batch size is set to 64, and the optimizer uses SGD. The experiments were deployed on a computer with Intel(R) Xeon(R) Gold 6246R CPU (3.4GHZ) and NVIDIA Quadro RTX 8000 GPU (48GB) having Windows 10 operating system with software configuration installed as Anaconda 3 -2021.11-windows version, using PyCharm compiler and given Pytorch1.2.1 built-in Python3.8.3 programming language, all the algorithms are run in the same environment.

## 3 Results and discussion

### 3.1 Impact of learning rate dynamic adjustment strategies on model

**Figure 6:**
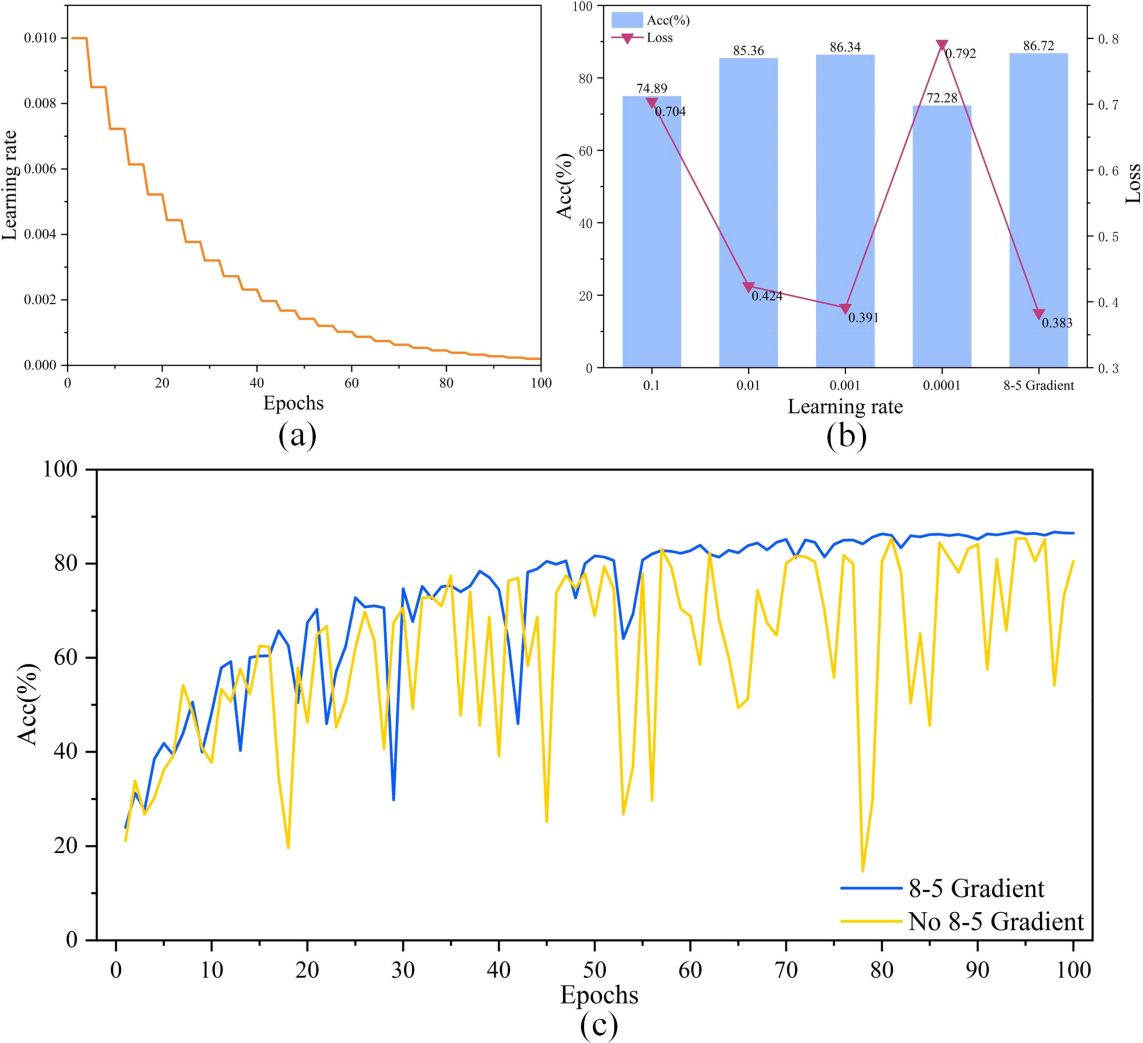
**(A)** is the trajectory of the learning rate driven by the “8-5 Gradient” during model training, **(B)** is the results of “8-5 Gradient” compared with other common learning rates, and **(C)** is the result of comparing the accuracy curves on the validation set after adding the 8-5 gradient and removing the 8-5 gradient.

To improve the performance of the model, this study first used FasterNet_T0 as the base model and optimized the parameters during the training of the model. As shown in **Figure 6A**, the trajectory of “8-5 Gradient” during the model training process is demonstrated, with the learning rate reduced every four rounds. **Figure 6B** shows that comparing “8-5 Gradient” with other representative learning rates, the results show that “8-5 Gradient” obtains the lowest loss and the highest accuracy, which reach 0.383 and 86.72%, respectively, ahead of the other learning rates. This result shows that “8-5 Gradient” has good performance.

The accuracy curves of adding an 8-5 Gradient and removing an 8-5 Gradient on the validation set are shown in **Figure 6C**. The accuracy of the model with an 8-5 Gradient removed fluctuates substantially during the training process, and the convergence and fitting cannot be completed. The model with 8-5 Gradient added, on the other hand, completes the convergence and fitting at the later stage of training, and the accuracy is further improved. This result further proves the advantage of an 8-5 Gradient.

### 3.2 Results of ablation experiments

This experiment delves into the multidimensional impact of model improvement strategies on overall model performance. The first attempt is to combine FasterNet_T0 with Ghost bottleneck, an improvement that significantly enhances the model’s representational capabilities, thus greatly improving the model’s accuracy from 86.78% to 91.13%. However, this enhancement does not come without a price, it also brings about a significant increase in computational effort, number of parameters, parameter size, and weight size. We replace 1×1Conv with 3×3GConv in the FasterNet_T0 backbone, which reduces the computation by 241.729M and makes the model more lightweight. It is worth noting that this optimization does not sacrifice performance, but instead slightly improves the accuracy of the model. This proves that GConv can significantly reduce the computational load and the number of parameters while maintaining performance. When both GConv and Ghost bottleneck are introduced, the accuracy is drastically improved by 5.22%, while the computational load is reduced by 90.957 M. The specific details of this improvement are shown in **Table 3**, which fully demonstrates the excellence of HPFasterNet in improving performance and reducing complexity.

**Table 3:** Evaluation results of the model in the ablation experiment.

Model	Acc(%)	P(Avg) (%)	R(Avg) (%)	F1(Avg) (%)	FLOPs(M)	Total Params (M)	Params Size(MB)	Weight Size(MB)
FasterNet_T0	86.78	87.12	86.86	86.92	339.389	2.649	10.1	20.3
FasterNet_T0+Ghost bottleneck	91.13	91.24	91.22	91.18	483.758	3.291	12.55	25.4
FasterNet_T0+GConv	88.61	88.73	88.67	88.63	97.666	1.029	3.93	7.95
**HPFasterNet**	**92.00**	**92.08**	**92.04**	**92.02**	**248.432**	**1.684**	**6.42**	**13.2**

Bold values represent the results of the algorithmic model proposed in this study.

### 3.3 Comparative experiments with other network models

Classical network models are popular for their high accuracy. Lightweight models are widely used in various mobile devices and embedded systems due to their smaller computational resource requirements. To validate the advantages of the network model HPFasterNet proposed in this study, three classical network models [ResNet50 (He et al., 2016), ConvNeXt_T (Liu et al., 2022), and RepVGGNet_A1 (Ding et al., 2021)] and three lightweight network models [GhostNet (Han et al., 2020), ShuffleNet (Zhang et al., 2018), and MobileNetV2 (Sandler et al., 2018)] were compared in this study. The results show that HPFasterNet has significant advantages over the classical network models. The FLOPs(M) of ResNet50, ConvNeXt_T, and RepVggNet_A1 are very large, reaching 4131.734, 4454.781, and 3428.012, respectively, and there is no significant advantage in recognition accuracy, reaching 90.09, 79.40, and 90.25, respectively, which are relatively HPFasterNet is lower. And when comparing other indicators, the classical network models all performed poorly. When comparing lightweight network models, HPFasterNet is slightly higher than ShuffleNet in time complexity and space complexity, but the recognition accuracy of the network model is 2.44% higher than ShuffleNet. Although GhostNet has lower FLOPs(M) relative to HPFasterNet, HPFasterNet has a clear advantage in other metrics. And when compared to MobileNetV2, HPFasterNet’s advantage is even more pronounced, leading MobileNetV2 in all metrics. Taken together, HPFasterNet obtains the optimal overall performance. The specific details are shown in **Table 4**.

**Table 4:** Evaluation results of HPFasterNet in comparison with other network models.

Model	Acc(%)	FLOPs(M)	Total Params (M)	Params Size(MB)	Weight Size(MB)	Time/Epochs
ResNet50	90.09	4131.734	23.547	89.82	179	112s
ConvNeXt_T	79.40	4454.781	27.813	106.16	212	141s
RepVGGNet_A1	90.25	3428.012	14.561	48.96	98.2	66s
GhostNet	89.08	154.607	3.926	14.98	30.2	61s
ShuffleNet	89.56	151.704	1.273	4.86	9.92	40s
MobileNetV2	90.53	326.295	2.248	8.58	17.4	62s
**HPFasterNet**	**92.00**	**248.432**	**1.684**	**6.42**	**13.2**	**60s**

Bold values represent the results of the algorithmic model proposed in this study.

**Figure 7:**
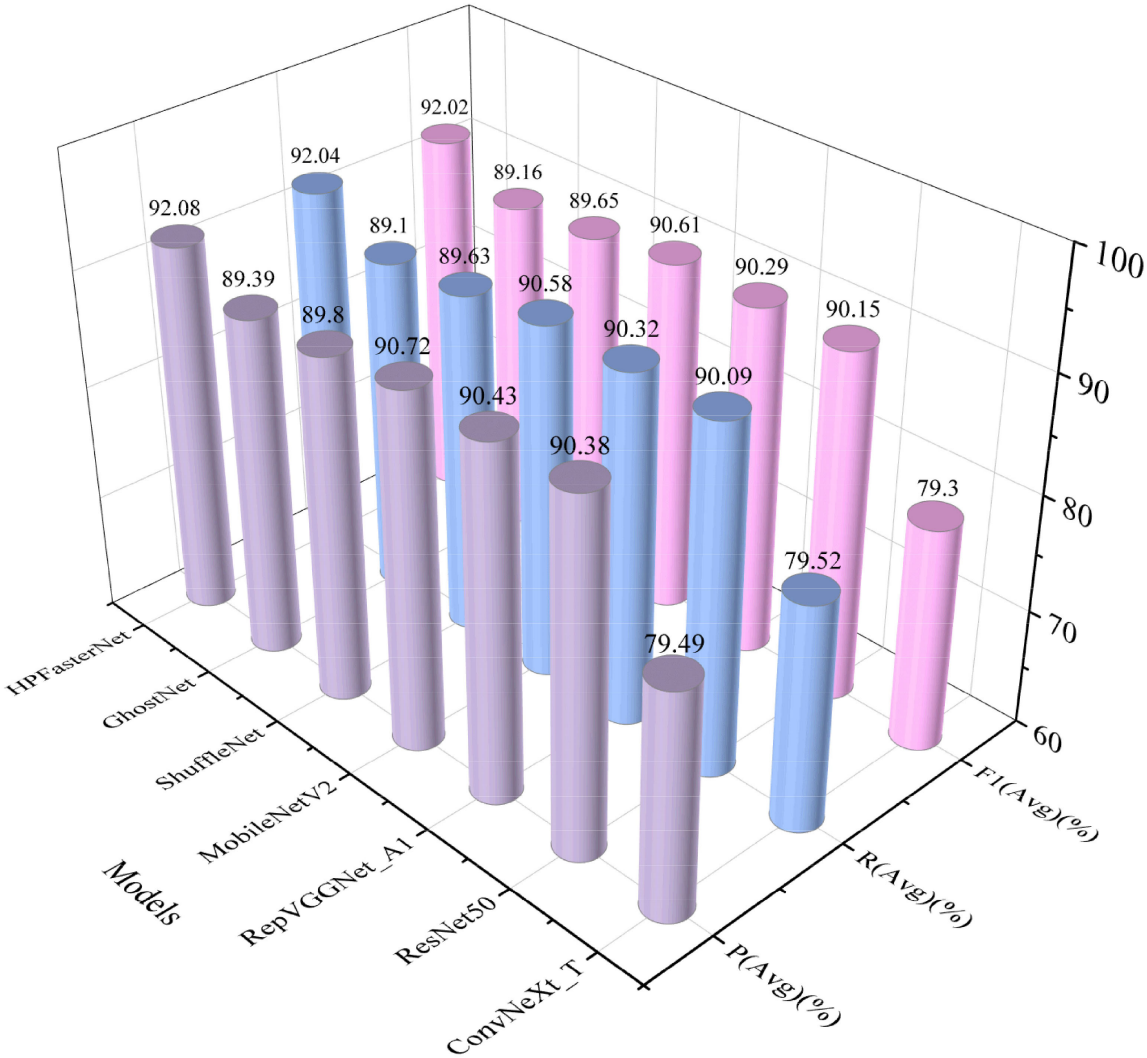
Comparison results of HPFasterNet with other network models in terms of precision, recall, and F1-score.

To further validate the advantages of HPFasterNet, the precision, recall, and F1 scores of the six network models were visually compared with HPFasterNet in this study, as shown in **Figure 7**. The precision, recall, and F1-scores of the six network models are not as good as HPFasterNet, with ConvNeXt_T having the worst performance, with precision, recall and F1-scores of 79.49%, 79.52% and 79.3%, respectively. Overall, among the six network models, HPFasterNet had the best recognition results.

### 3.4 Before and after model improvement

**Figure 8:**
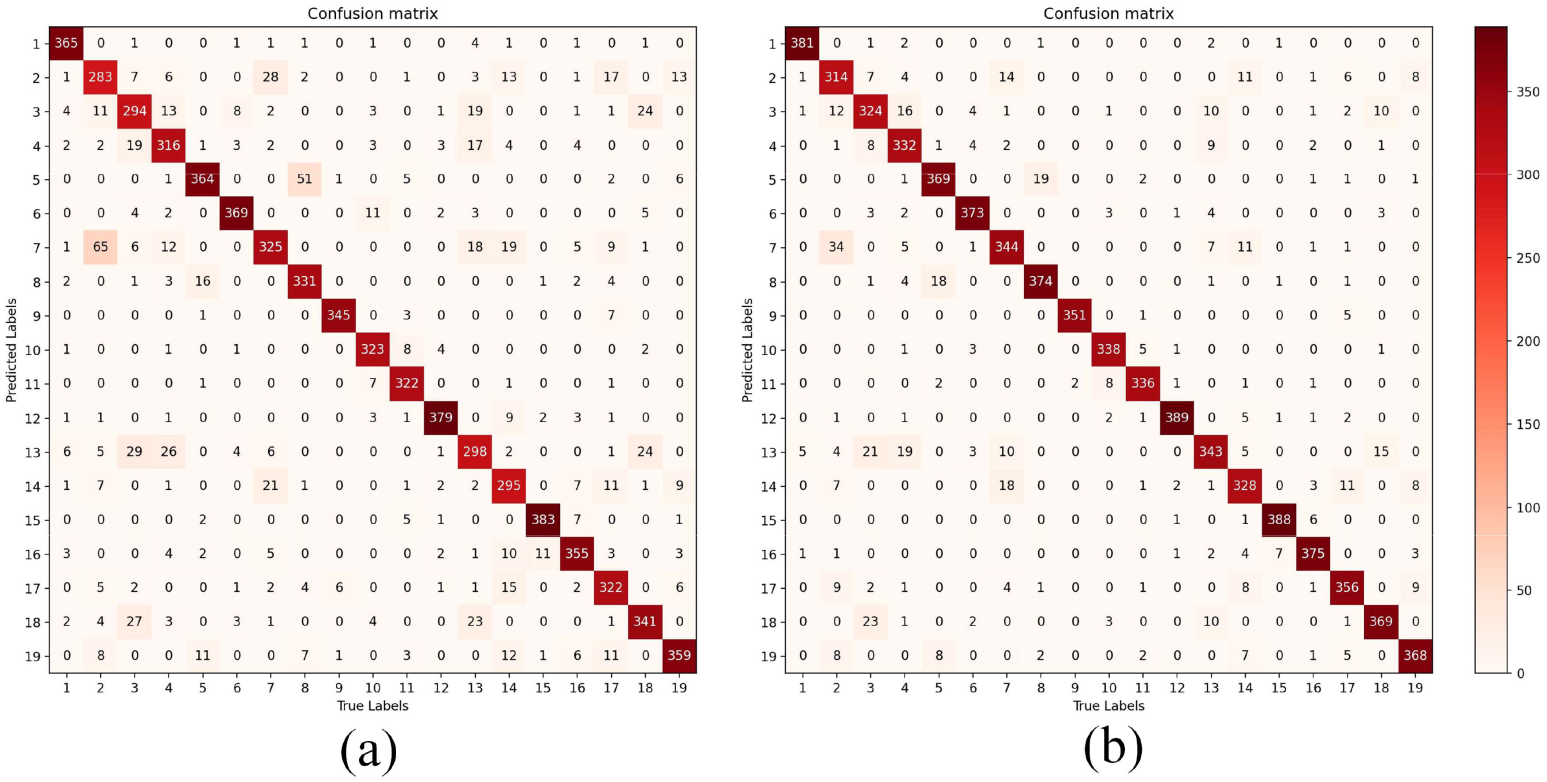
Confusion matrix before and after model improvement, **(A)** is the confusion matrix of FasterNet_T0 in the test set, **(B)** is the confusion matrix of HPFasterNet in the test set.

To evaluate the performance of the model more comprehensively, the comparative analysis of the confusion matrix before and after the model improvement was plotted in this study to reflect the actual recognition of each variety by FasterNet_T0 and HPFasterNet. As shown in **Figure 8**, before the improvement, due to the limited ability of FasterNet_T0 for feature extraction and the similarity of features among different varieties of rice, the error of some varieties is large, such as H003 and H008, which is easy to misclassify. While the improved model can reduce the misclassification to a certain extent, the values on the diagonal line are also significantly improved with darker colors, which indicates that the recognition rate of the model on each category has been significantly improved. In addition, the overall structure of the confusion matrix has become clearer, with a higher degree of differentiation between categories. Overall, the improved model demonstrated better performance on the classification task, providing more reliable and accurate classification results for related applications.

As shown in **Table 5**, based on the confusion matrix, a comparison of each category before and after the improvement in terms of precision, recall, and F1 score can be derived. The precision before improvement varies widely between categories, and it is worth noting that certain categories have relatively low precision. After model improvement, the precision of all the categories is improved, especially on the categories that had lower precision before. For example, on H032, the accuracy before the improvement was 70.5%, while after the improvement it increased to 85.1%. This means that 85.1% of the samples predicted by the model as H032 belong to H032, a significant improvement over the 70.5% before the improvement. Similarly, for each category, the recall before improvement varies. After the model improvement, the recall of all categories is improved, especially on the categories with lower recall than before, the improvement is more significant. In the case of H114, for example, the recall before improvement is 76.6%, while after improvement it increases to 88.2%. This means that 88.2% of all samples that belong to H114 are correctly predicted as H114 by the model, which is a significant improvement compared to 76.6% before the improvement. After model improvement, the F1-scores of all categories are improved, especially on the categories with lower F1-scores before, the improvement is more significant. In the case of H008, for example, the F1 score before the improvement is 73.9%, while after the improvement it increases to 83.0%. This indicates that the comprehensive performance of the model on H008 has been effectively improved, both in terms of precision and recall. The experiments show that after the model improvement, the performance of all the categories is improved in terms of precision, recall, and F1 score. This indicates that the classification ability of the model has been effectively enhanced to better accommodate the differences between the different categories.

**Table 5:** Analysis of results for each category before and after model improvement.

Label	Variety	P (%)		R (%)		F1 (%)	
		Before	After	Before	After	Before	After
1	H005	96.8	98.2	93.8	97.9	95.3	98.0
2	H008	75.5	85.8	72.4	80.3	73.9	83.0
3	H009	77.2	84.8	75.4	83.1	76.3	83.9
4	H024	84.0	92.2	81.2	85.3	82.6	88.6
5	H029	84.7	93.7	91.5	92.7	88.0	93.2
6	H030	93.2	95.9	94.6	95.6	93.9	95.7
7	H032	70.5	85.1	82.7	87.5	76.1	86.3
8	H103	91.9	93.5	83.4	94.2	87.4	93.8
9	H105	96.9	98.3	97.7	99.4	97.3	98.8
10	H109	95.0	96.8	91.0	95.2	93.0	96.0
11	H110	97.0	95.7	92.3	96.3	94.6	96.0
12	H113	94.5	96.5	95.7	98.2	95.1	97.3
13	H114	74.1	80.7	76.6	88.2	75.3	84.3
14	H156	82.2	86.5	77.4	86.1	79.7	86.3
15	H179	96.0	98.0	96.2	97.5	96.1	97.7
16	H184	89.0	95.2	90.1	95.2	89.5	95.2
17	H186	87.7	90.8	82.4	91.0	85.0	90.9
18	H230	83.4	90.2	85.5	92.5	84.4	91.3
19	H242	85.7	91.8	90.4	92.7	88.0	92.2

**Figure 9:**
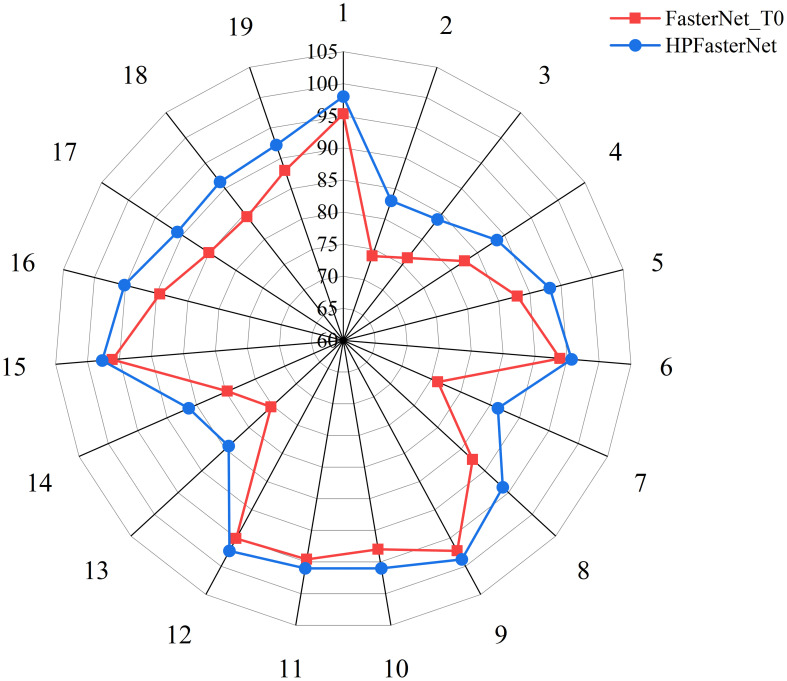
Comparison of F1-scores on each category before and after model improvement.

The F1-score is the reconciled average of precision and recall, which is used to comprehensively evaluate the performance of the model. Before improvement, the F1-score varies as precision and recall vary between categories. To further assess the comprehensive performance of the model before and after the model improvement, this study visualized and compared the model’s F1-scores in each category, as shown in **Figure 9**. From the figure, it can be seen more clearly that the F1-scores between the categories of the model before the improvement are more different and the model has a poorer recognition ability after the model improvement is completed, the F1-scores of each category are improved, and presents a more balanced distribution, and the differences between the categories become smaller. This result indicates that the overall performance of the model has been significantly improved, and the adaptability and generalization ability of the model in each category has been enhanced. For some categories with lower recognition accuracy before improvement (e.g., category 2, category 3, category 7, and category 13), the model gives more attention and optimization, which results in a significant increase in their recognition accuracy after improvement, so that the overall performance of the model as a whole has been substantially improved.

**Figure 10:**
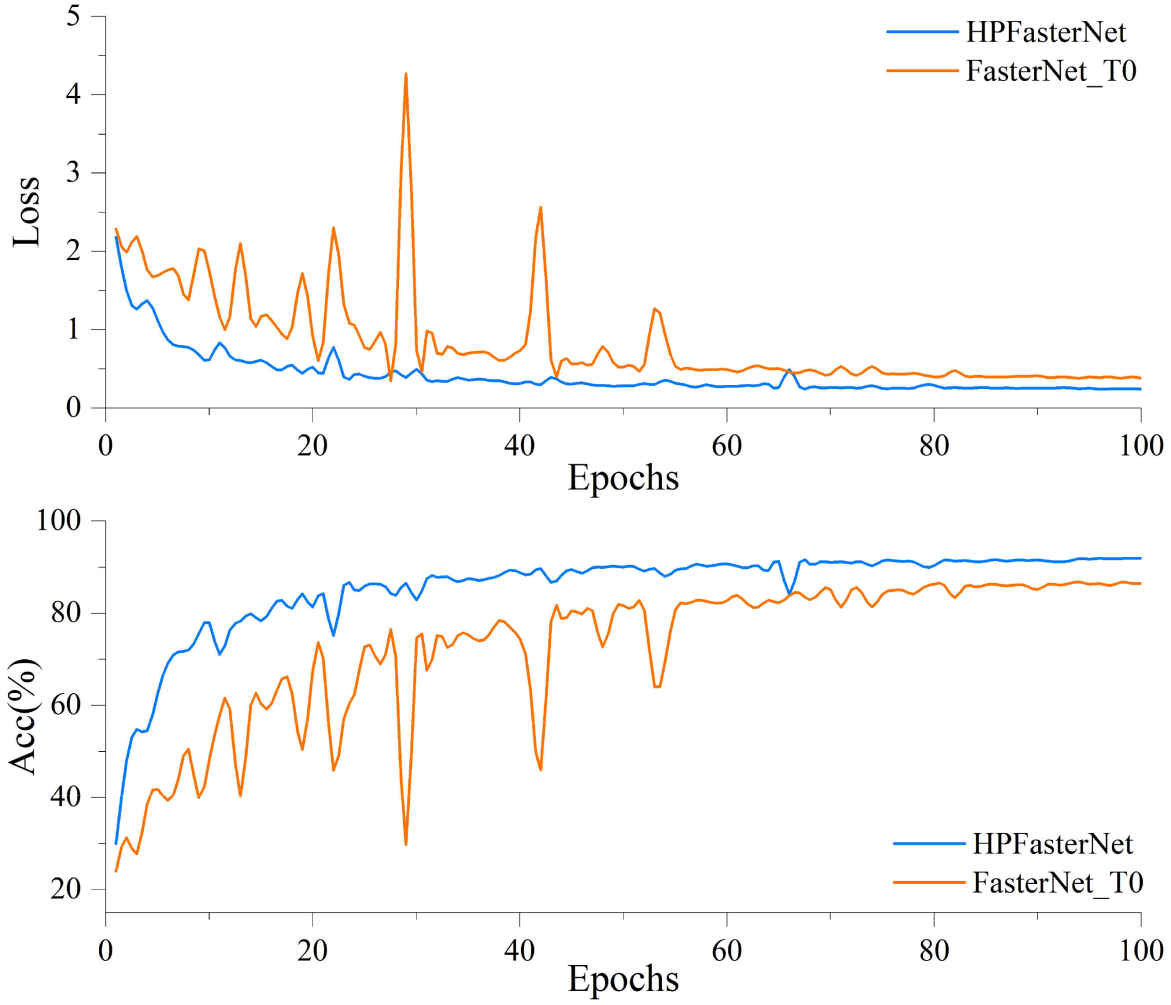
Comparison of loss curves and accuracy curves on the validation set before and after model improvement.

**Figure 10** shows the loss curve and accuracy curve on the validation set before and after the model improvement. The accuracy curve before model improvement still has large fluctuations in the pre-training period, but then the growth rate gradually slows down and stabilizes at the end of the training period. Although a relatively stable level of accuracy can eventually be achieved through the 8-5 Gradient model, the model still has large fluctuations in the early stages of training, and there is an overall risk of overfitting. In contrast, the accuracy curve of the improved model shows a more obvious upward trend, and the accuracy rises rapidly in the early stage of training, and then continues to maintain a stable growth trend, eventually reaching the highest accuracy level and stabilizing. Meanwhile, the loss curve of the model before improvement fluctuates greatly in the early stage of training, but gradually flattens out in the late stage of training, indicating that the model encounters optimization difficulties during training and the loss is difficult to be further reduced. In addition, the higher final value of the loss curve indicates that the model has limited fitting ability and may have overfitting or underfitting problems, while the improved loss curve shows a more desirable downward trend. Ultimately, the smaller the value of the loss curve, means that the fitting ability of the model is significantly improved and can better adapt to the training data. By comparing the loss curves and accuracy curves before and after the improvement, we can see that the optimization ability and accuracy of the improved model have been significantly improved.

### 3.5 Test results of rice seeds with different flavors

**Figure 11:**
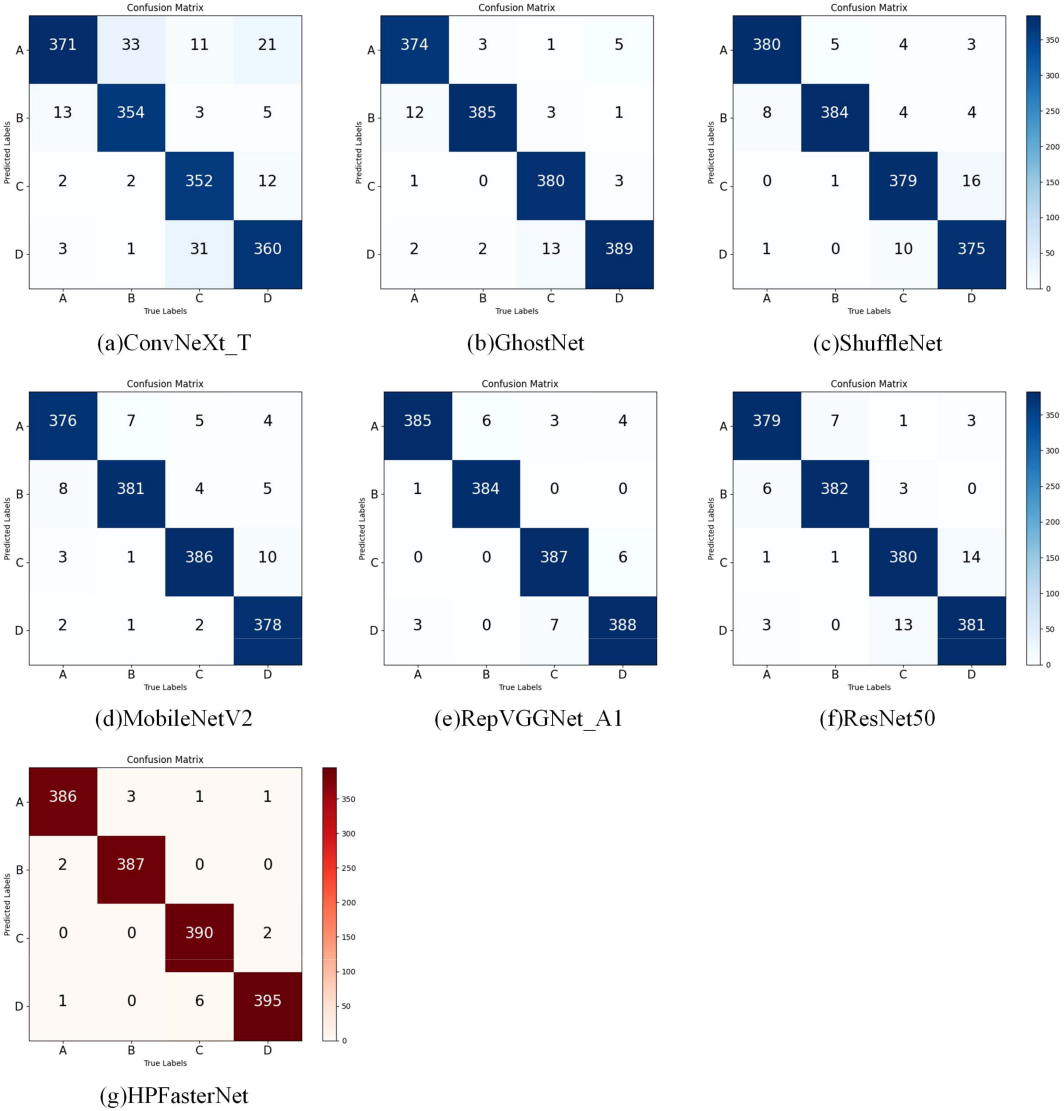
Comparison of confusion matrices for different models testing different flavors of rice.

The following experiments were done to verify the effectiveness of the network model proposed in this study for the recognition of rice with different flavors. As shown in **Figure 11**, comparing the confusion matrices of the models in the experiment, when comparing the confusion matrices of the other six models, it can be noticed that each model performs differently on the classification task. Looking at the confusion matrix of HPFasterNet, we can see that the values on the diagonal line are relatively high, which means that the model performs well in correctly classifying flavored rice. The higher values on the diagonal line indicate that the model has a higher prediction accuracy for the corresponding category. Meanwhile, relatively low values on the off-diagonal line mean that the model misclassified samples to other categories less often. In contrast, the confusion matrices of the other six models show different degrees of variation. The lower values on the diagonal of the confusion matrices of the other models relative to HPFasterNet indicate that they are not as accurate as HPFasterNet on the classification task. This result suggests that the improved model has a clear advantage in classification performance.

The accuracy, precision, recall, and F1-scores of each network model on different flavored rice were derived from the confusion matrix, and as shown in **Table 6**, the accuracy, precision, recall, and F1-scores of HPFasterNet reached 98.98%, 99.00%, 98.95%, and 98.93%, respectively. The results show that HPFasterNet outperforms the other six network models, and it can be concluded that the network model proposed in this study shows excellent performance in flavored rice recognition.

**Table 6:** Comparative experimental results of the model on flavored rice.

Models	Acc(%)	P(%)	R(%)	F1(%)
ConvNeXt_T	91.30	91.58	91.35	91.38
GhostNet	97.08	97.13	97.05	97.05
ShuffleNet	96.44	96.45	96.48	96.45
MobileNetV2	96.70	96.70	96.70	96.68
RepVGGNet_A1	98.09	98.10	98.13	98.10
ResNet50	96.70	96.73	96.68	96.68
**HPFasterNet**	**98.98**	**99.00**	**98.95**	**98.93**

Bold values represent the results of the algorithmic model proposed in this study.

## 4 Conclusions

This study delves into the application of the improved lightweight network HPFasterNet in the field of rice, especially in classifying and recognizing rice seeds of different varieties. We make important improvements to the traditional FasterNet_T0 model by introducing efficient feature extraction modules Ghost bottleneck and GConv. These innovations not only significantly improve the model’s accuracy in classifying rice seed grains by 5.22%, but also dramatically reduce the computational complexity and the number of parameters, making HPFasterNet an ideal choice for resource-constrained environments. In the comparison experiments, we selected three classical network models and three lightweight network models as references. Through exhaustive performance evaluation and comparative analysis, we find that HPFasterNet demonstrates significant advantages in several key metrics. HPFasterNet can accurately distinguish rice seeds of different varieties, which is mainly attributed to its powerful feature extraction capability and optimization strategy. This feature information is fully utilized by the model, thus achieving high-precision and fast classification recognition. The experimental results also show that HPFasterNet can accurately capture the subtle differences between different flavored rice, and can accurately differentiate between different flavored rice with an accuracy of 98.98%.

However, despite the results achieved in this study, we are aware of some limitations. First, due to the diversity of rice varieties and the complexity of growing environments, existing feature extraction and classification methods may not be able to fully cover all situations. Although HPFasterNet has adopted advanced techniques such as partial convolution (PConv) to improve performance, more efficient network structures, such as the introduction of attention mechanism, deformable convolution, etc., can be further explored in the future to enhance the model’s ability to extract features from rice seeds. To meet the real-time and low-power requirements in practical applications, it is possible to investigate how to further reduce the size and computational complexity of the model with guaranteed accuracy, e.g., through model pruning and quantization. Consider combining rice seed classification with other related tasks (e.g., seed counting, pest and disease detection, etc.) to improve the model’s generalization ability and practicality through multi-task learning. To improve the performance and generalization ability of the algorithm, it is necessary to construct a larger and more diverse rice seed dataset, including seed images of different varieties, different growth stages, and different light conditions.

HPFasterNet can be used for crop monitoring and management in precision agriculture, e.g., by real-time monitoring of rice growth and seed yield, it can provide farmers with precise suggestions for irrigation and fertilization. Combined with other image processing technologies, HPFasterNet can be further applied to early warning and control of pests and diseases to improve crop yield and quality. In the food processing industry, HPFasterNet can be used to classify and detect food ingredients, such as distinguishing different varieties of rice, to ensure the quality and safety of food. By recording and analyzing key information in the food production process, HPFasterNet can assist in realizing food traceability and tracking, and improve the efficiency of food safety management. In ecology, HPFasterNet can be used to categorize and monitor plant populations, helping to understand changes and trends in biodiversity. By monitoring and analyzing plant growth, HPFasterNet can indirectly reflect the health of the environment and provide the scientific basis for environmental protection and governance.

## Data Availability

The original contributions presented in the study are included in the article/supplementary material. Further inquiries can be directed to the corresponding author.
